# Radiolabeling Strategies for Tumor-Targeting Proteinaceous Drugs

**DOI:** 10.3390/molecules19022135

**Published:** 2014-02-18

**Authors:** Grant Sugiura, Helen Kühn, Max Sauter, Uwe Haberkorn, Walter Mier

**Affiliations:** Department of Nuclear Medicine, University Hospital Heidelberg, Im Neuenheimer Feld 400, Heidelberg D-69120, Germany

**Keywords:** radionuclides, radiometals, chelator, prosthetic groups, radiohalogenation, carrier molecules, proteins, radiopharmaceuticals, diagnostic imaging

## Abstract

Owing to their large size proteinaceous drugs offer higher operative information content compared to the small molecules that correspond to the traditional understanding of druglikeness. As a consequence these drugs allow developing patient-specific therapies that provide the means to go beyond the possibilities of current drug therapy. However, the efficacy of these strategies, in particular “personalized medicine”, depends on precise information about individual target expression rates. Molecular imaging combines non-invasive imaging methods with tools of molecular and cellular biology and thus bridges current knowledge to the clinical use. Moreover, nuclear medicine techniques provide therapeutic applications with tracers that behave like the diagnostic tracer. The advantages of radioiodination, still the most versatile radiolabeling strategy, and other labeled compounds comprising covalently attached radioisotopes are compared to the use of chelator-protein conjugates that are complexed with metallic radioisotopes. With the techniques using radioactive isotopes as a reporting unit or even the therapeutic principle, care has to be taken to avoid cleavage of the radionuclide from the protein it is linked to. The tracers used in molecular imaging require labeling techniques that provide site specific conjugation and metabolic stability. Appropriate choice of the radionuclide allows tailoring the properties of the labeled protein to the application required. Until the event of positron emission tomography the spectrum of nuclides used to visualize cellular and biochemical processes was largely restricted to iodine isotopes and 99m-technetium. Today, several nuclides such as 18-fluorine, 68-gallium and 86-yttrium have fundamentally extended the possibilities of tracer design and in turn caused the need for the development of chemical methods for their conjugation.

## 1. Introduction

Proteinaceous drugs, also referred to as biologics, have broken into clinical routine. The current rate of medical advancement demands information regarding the molecular events and mechanisms of new chemical entities. This is a new area of analytics as the pharmacokinetic behavior of proteins differs from that of the small drugs of a molecular weight that correspond to the traditional understanding of druglikeness. The methods available to analyze the pharmacokinetics and metabolism have to keep step with this tremendous development. The sensitivity at which radioisotopes allow noninvasive tracing of tiny amounts of drugs perfectly matches with the challenges posed by the visualization of the pharmacokinetics of the effectors used in new treatment strategies such as gene therapy and therapeutic vaccination. 

By incorporating radioisotopes into molecules designed specifically for a target, it is possible to follow a drug’s journey in man. Imaging modalities, such as SPECT and PET, offer high sensitivity to such radionuclides. Proteins possess a unique structure key to target specificity making them perfect vehicles for radioisotopes. However any alteration to such a biomolecule must ensure that it does not affect the biological activity of the structure. Thus, techniques used in the incorporation of a radioisotope must be biocompatible with the labeled molecule. The techniques used must be able to provide good yields, stability and an unaltered bioactivity of the products. In the case of proteins the task to stably bind radioisotopes to the molecule to be studied differs from the methods used for small molecules. While small molecules have to be labeled with ^14^C and ^1^H to ensure their function, proteins will accept stronger modifications. The two main methods of modification of proteins are halogenation and the complexation of metallic radionuclides. The need for new and innovative techniques have led to the use of chelating agents as indirect methods of incorporation for radioisotopes plus the improvement of direct labeling approaches. Having an understanding of the intricate reactions which occur *in vivo*, will allow for the precise determination of a pharmaceutical’s pharmacology.

The radiolabeling of molecules for clinical use has developed a significant benefit. The use of radionuclides in molecular imaging provides vital data on the characteristics of new drugs *in vivo*. The ability to analyze a drug’s pharmacokinetics in man will provide important safety and dosing information. The development of ‘first in man studies’ with radiation involves low doses that present little risk. The combination of this with proteinaceous targeting systems would provide invaluable knowledge about molecular targets and protein expression. This precise stratification can be achieved with radioactive labeled antibodies.

Small molecules can be labeled with ^14^C as a radioisotope. However this presents two drawbacks: (i) ^14^C is a β-emitter, unfavorable in molecular imaging and (ii) it has a long half-life of 5,730 years, which results in large levels of irradiation. An alternative would be to use ^11^C, which possesses a half-life of 20.33 min and provides the possibility to conduct positron emission tomography. Carbon nuclides are useful in small organic molecules, however they will be of little benefit in macromolecular proteins, *ergo* different nuclides must be used. With proteins, any major modification may cause an alteration in biological activity. However minor modifications such as chelation or halogenation often allow the protein to retain its biological properties/activity. Therefore the use of radionuclide-chelating agents or radiohalogens could be key in the development in these innovative studies. 

**Figure 1:**
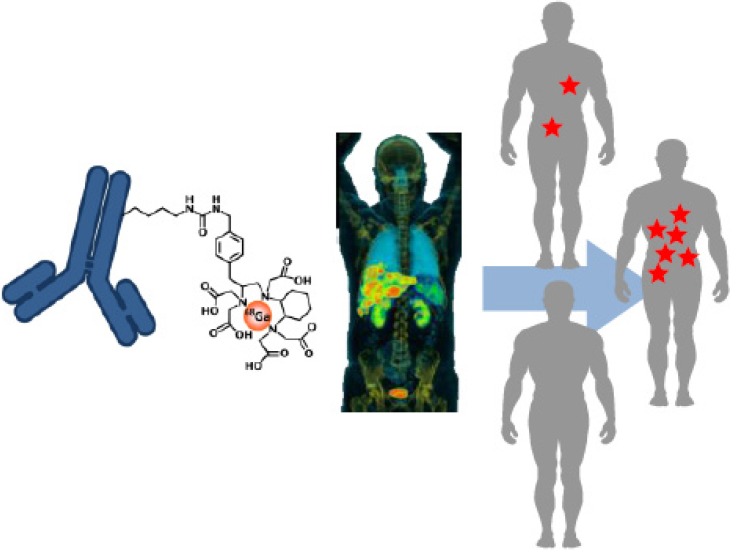
Illustration showing the use of a radiolabeled antibody in personalized medicine. The introduced radiopharmaceutical allows for an indication of which patients will respond better to therapy, due to the heterogeneity of tumors, thus suggesting radiolabeled antibodies have potential use in stratified medicine.

First in man studies are of utmost importance in ‘personalizing’ medicine. The tailoring of a medication regime to an individual patient is the clinical therapeutic model known as personalized medicine. Personalized medicine goes further than prescreening as it also takes into account patient dependent efficiency and tolerance to chemotherapeutic side effects (Figure 1). Personalizing a medication regime can evaluate what dose of a drug will be effective in a specific patient. In combination with the possibility of using the target specificity of proteinaceous drugs, personalized medicine can widen the therapeutic window of anti-tumor medication.

## 2. Molecular Imaging

In modern medicine, not only the detection of tumors but also their molecular characterization is sought after and considered significant in the diagnosis and prognosis of tumors. Molecular imaging therefore represents an emerging medical field. It allows the visualization, characterization and quantification of tumors and biological processes using probes known as molecular imaging agents, such as radionuclides or fluorescent probes. The benefits of molecular imaging include the characterization of heterogeneity of tumor receptor expression, detection of molecular targets for personalized therapy and the noninvasive nature of the field [1]. Molecular imaging is of even greater importance for tumors not easily accessible by other methods such as thyroid and recurrent intrahepatic tumors [2,3]. There are currently five main imaging techniques used clinically: CT, ultrasound, MRI, SPECT and PET. With the innovative introduction of 3D imagery four of these techniques, CT, MRI, SPECT and PET, are able to generate a 3-dimensional detection around the body. However, the limits of detection of these techniques are around 10^9 ^cells (corresponding to approximately 1 gram of tissue). This results in patients and cancers which are considered in a state of remission potentially possessing a large, undesirable and uncertain number of cancerous cells. As well as this, the elevated detection range limits the early detection and the effectiveness of small metastases recognition [4]. 

In tumor cells it is common for a particular receptor to be overexpressed and in abundance. For molecular imaging this overexpression provides a useful clinical target, for example CD20 receptors in Non-Hodgkin’s lymphomas, a validated target for anti CD20 antibodies. It is important to consider however that even small concentrations of imaging agents may result in saturation of receptors causing increased nonspecific background binding. Therefore minute molar quantities of imaging agents are required. This has resulted in the radionuclide imaging methods PET and SPECT being preferred due to the micro- to picomolar concentrations used. [1] A variation of this is imaging with antibodies, known as radioimmunodetection, involves the labeling of specific antibodies for detection with radionuclides. This enables the detection of these labeled antibodies by highly sensitive imaging methods such as SPECT and PET. In single-photon emission computed tomography (SPECT) a gamma emitter is introduced to the patient and the emitted gamma rays are measured directly. The most commonly used radionuclide in SPECT is ^99m^Tc, decaying mainly by gamma emission, yet a small fraction also decays by internal conversion. Besides detection and imaging of tumors, there have been numerous advancements in SPECT imaging for determining response to therapy, in particular by use of ^99m^Tc-labeled recombinant human Annexin V [5].

Conversely, PET imaging utilizes positrons, emitted by proton rich nuclei, which annihilate with electrons of molecules in surrounding tissue. The interaction of this positron and electron creates two gamma photons, produced at a 180 degree angle to each other and detectable by a PET scanner. PET scanners are becoming more significant in clinical utility due to the higher resolution images produced compared to SPECT. Thus, PET is now considered a vital tool in the imaging of tumors. One area where PET is proving extremely beneficial is the staging of cancerous tumors, as a result of the increased accuracy achieved with PET [6]. ^18^F is the most commonly used radionuclide for PET imaging owing to its half-life of 109.8 mins (see Table 1). This half-life allows for synthesis and delivery from an external site to the PET center and finds a balance to allow for a minimized dose of radioactive substance to a patient [7]. It also has a low energy positron emission which decreases the risk for patients, which also leads to a lower linear range in tissue, enabling a higher spatial resolution image in PET [8]. Furthermore carbon-fluoride bonds are similar in length to carbon-oxygen bonds which produce reasonable bioisosterism, improving the binding potential of radiotracers where hydroxyl functional groups are substituted for ^18^F [9]. ^18^FDG, an analogue of glucose, provides information in regions of high metabolic glucose activity, such as tumors. ^18^FDG is the most commonly used radiotracer clinically and is generally considered the gold standard of radiopharmaceuticals for oncological PET imaging. However with the advancement of current medical knowledge, there is a requirement for more detailed biochemical event knowledge. This can be provided by use of other radionuclides and PET imaging drugs. Other radionuclides which are also utilized are the metallic nuclides, such as ^64^Cu, ^67/68^Ga and ^86^Y used in peptide and protein radiotracers. 

**Table 1:** Radionuclides involved with the radiolabeling of proteins and their mode of decay. For imaging purposes, the release of γ-rays are preferred either by positron emission, Electron capture (EC) or internal conversion (IC).

Radionuclide	Mode of Decay	Half-life	Emax (mean)
^18^F	β^+^	109.8 min	0.63 MeV
^68^Ga	β^+^	68 min	1.90 MeV
^99m^Tc	IC	6.02 h	0.14 MeV
^111^In	EC	2.8 d	0.24 MeV
^123^I	EC	13.2 h	0.16 MeV

Technological advancements in PET imaging have further confirmed the significance of this imaging technique. Methods such as time of flight reduce the statistical noise in the reconstructed images by determining the exact location of annihilation along the detected line of response [10]. Other developments include positron emission mammography, which provides high specificity and resolution images of early *in situ* breast cancer. PET and SPECT enable the precise imaging of tumor sites provided there is a large enough expression of the targeted receptor, often insufficient in tumors in a state of remission, as mentioned earlier. Due to tumor heterogeneity, cancerous sites lacking the desired feature are overlooked. For this reason combination imaging modalities such as PET/CT or PET/MRI are often utilized. The development of multimodal imaging agents has become an important challenge. Targeted peptides and antibodies are important vectors for molecular imaging [11]. Antibodies exhibit high affinity and contact time to cell surface antigens for *in vivo* quantification and tracking of antibodies. The long elimination half-life of antibodies requires a radionuclide with a long half-life, such as ^123^I, in order to maximize efficacy between the antibody-radionuclide complex (see Table 2).

**Table 2:** The four isotopes of iodine currently in use in radiochemistry and their applications.

Isotope	Half-life	Application
^123^I	13.2 h	SPECT imaging
^124^I	4.2 days	PET imaging
^125^I	59.4 days	Small animal imaging & *in vivo* studies
^131^I	8.0 days	SPECT imaging & therapy

## 3. Radiotherapy

Besides using radiolabeled drugs to specifically image tumors, radionuclides also possess importance in the therapy of cancers as is evident by the number of antibody-based drugs in clinical practice (see Table 3). Endoradiotherapy utilizes the specific targeting obtainable with tracers including peptides and proteins and combines it with the powerful radiation induced cell death capabilities of radionuclides. These therapeutics are capable of being labeled with either a therapeutic or diagnostic nuclide. The benefit of using a radionuclide is that neither the drug nor antibody needs to be internalized to exert its therapeutic effects, due to the cross fire effect of high energy β-emitters. This results in smaller quantities of drugs being required to bring about the desired therapeutic effects [12]. The main criteria in determining the success of an endoradiotherapeutic as well as a diagnostic agent is the stability of the radionuclide attached. A labeled agent with low stability will have diminished target specificity and a greater potential harm to normal tissues, due to dissociation of the radionuclide. The stability of a radiopharmaceutical drug is usually analyzed in human serum and measured using radio-TLC, radio-HPLC and LC/MS. These techniques are based on the chromatographic separation of the radiolabeled compounds and their subsequent evaluation [13].

**Table 3:** A selection of clinically used/trialed antibody based drugs, their targets and, if applicable, method of radiolabel attachment.

Name	Protein	Antibody Form	Radionuclide attached	Target	Application	Clinical Trial	Method of Attachment
Rituximab	Anti-CD20 mAB	Chimeric	N/A	CD20	B-cell non-Hodgkin’s lymphoma	FDA approved	N/A
Trastuzumab	Anti-HER2 mAB	Humanized	N/A	HER2	HER2 positive breast cancer	FDA approved	N/A
Pertuzumab	Anti-HER2 mAB	Humanized	N/A	HER2	HER2 Positive breast cancer	FDA approved	N/A
Avastin^®^ (bevacizumab)	Anti-VEGF mAB	Humanized	N/A	VEGF-A	Angiogenesis inhibitor	FDA approved	N/A
Zevalin^®^ (ibritumomab)	Anti-CD20 mAB	mu IgG1	^90^Y	CD20	B-cell non-Hodgkin’s lymphoma	FDA approved	MX-DTPA
Bexxar^®^ (tositumomab)	Anti-CD20 mAB	mu IgG2a	^131^I	CD20	B-cell non-Hodgkin's lymphoma	FDA approved	Iodination
Epratuzumab	Anti-CD22 mAB	Humanized	^90^Y	CD22	B-cell non-Hodgkin’s lymphoma	Phase I/II	DOTA
Veltuzumab	Anti-CD20 mAB	Humanized	N/A	CD20	B-cell non-Hodgkin’s lymphoma	Phase I/II	N/A
Clivatuzumab	Anti-MUC1	Humanized	^90^Y	MUC1	Pancreatic cancer	Phase I/II	DOTA

A very relevant field is radioimmunotherapy (RIT) using antibody-based labeled drugs. It combines the synergistic effects of radio- and immunotherapy and is an important tool in the treatment of hematologic malignancies such as Non-Hodgkins lymphoma [14,15]. Like standard chemotherapy, RIT has been optimized and standardized as a therapy for lymphomas, although not for solid tumors due to its low penetration rate and lower radiosensitivity. Studies have shown that the larger the tumors the less efficacy RIT will have. This is due to the fact that antibodies lack sufficient penetrative capabilities to reach the center of a large tumor mass. Tumors larger than 3–4 cm in diameter currently cannot be addressed with endoradiotherapy, with the most benefit possible in combination with subtherapeutic chemotherapy doses [16,17]. Large doses of radiolabeled antibodies are required to obtain any significant penetration required for these large solid tumors. Such large quantities prove troublesome due to the low blood clearance of radiolabeled antibodies, leading to the potential exposure of radiation sensitive tissues to the radionuclides [18].

## 4. Radionuclides

The main effect of endoradiotherapeutic agents is caused by the cytotoxic effects of radionuclides. Ionizing radiation is emitted by radionuclides and is utilized in endoradiotherapy for ionization and free radical formation. This emitted radiation results in subcellular damage by the energy deposition on cells during transversion, inducing cell death [19].

The specific cytotoxic effects of an agent on the cancer tissue are dependent on the physical properties of the radionuclide. The half-life of the radionuclide, its mode of decay and correspondingly the linear transferred energy, as well as the rate of energy deposition, are all properties with an influence on efficacy. Therefore the choice of appropriate radionuclide is of vital importance [18]. Other factors to consider when selecting a radionuclide include tumor uptake and retention, blood clearance, rate of radiation delivery, and the feasibility of large-scale production of the radionuclide in an economical fashion. Besides the direct radiation induced effects, radiation also causes indirect, so called bystander effects, on non-radiated cells. These bystander effects are mediated by gap-junction intracellular communication and through irradiated cell secretion of factors, inducing transduction pathways [20].

Therapeutic radiopharmaceuticals use particulate radiation such as α- and β-particles, yet also Auger electrons. Αlpha-radiation exhibits a high localized energy deposition combined with a short tissue range. These characteristics lead to a high Linear Energy Transfer (LET [21]. Thus α-radiation is the most damaging particulate radiation. Furthermore it can be applied in small concentrations since only 1-4 α-particles need to hit the cell to induce cell death [22]. As such, α- radiation is often used in residual tumors and hematological diseases, such as leukemia [23]. Alpha particles possess tissue ranges of less than a cell diameter. Consequently no cross-fire occurs. On the one hand this reduces the potential for unwanted side-effects on neighboring healthy tissue but increases the requirement for effective cell penetration of the therapeutic [24].

Due to this obstacle in therapeutic design, radionuclides for therapy are preferentially β-particle emitters (see Table 1). Compared to α-emitters, particulate β-emitters have a lower toxicity due to a lower deposition energy. In contrast to α-emitters, their therapeutic effect is mainly caused by the cross-firing bystander effects. This is due to β-emitters possessing a penetration range of several millimeters. Thus even cancerous cells located in the inner mass of tumors can be affected by the radiation, even if penetration rate of the pharmaceutical is poor. For example, long range β-emitters like ^90^Y and ^188^Re are used in the therapy of large tumor masses, due to their excellent penetration capabilities. [25] Conversely, a high penetration also accommodates an increased likelihood of side-effects in healthy cells [26].

In comparison to nuclides used for therapy, imaging agents use non particulate emitters such as γ-ray and positron emitters (Table 4). This is due to their low LET and rate of cell damage. The gamma emission resulting from electron capture (EC) or internal conversion (IC), results in the emission of Auger electrons. These Auger electrons traverse a very short range of only a few nanometers. Within these an energy deposition takes place, which is significantly higher than that of β- emitters. This results in a high LET which can be highly damaging when the emission occurs close to the DNA. Studies have shown that auger electrons, attached to carriers targeting nuclear DNA, cause a monoexponentional decrease in cell survival [22].

**Table 4:** List of radionuclide currently used in radiotherapy and their nuclear properties. As visible, all decay via α- or β-particulate emissions.

Radionuclide	Mode of Decay	Half-life	E_av_	Mean Tissue Range
^90^Y	β	2.7 d	2.27 MeV	2.76 mm
^131^I	β,γ	8.0 d	0.61 MeV	0.40 mm
^177^Lu	β, γ	6.7 d	0.50 MeV	0.28 mm
^225^Ac	α, β	10.0 d	6.83 MeV	0.04–0.1 mm
^213^Bi	α	47.5 min	8.32 MeV	0.04–0.1 mm
^212^Bi	α	1.0 h	6.21 MeV	0.04–0.1 mm
^211^At	α	7.2 h	6.79 MeV	0.04–0.1 mm
^212^Pb	β	10.6 h	0.57 MeV	0.6 mm

## 5. Proteins as Site-Specific Drugs

Proteinaceous drugs have gained importance as reflected by their impressive advent among the top pharmaceutical products [27]. The size of therapeutically used protein formats can vary significantly. While small proteins with molecular weights ≤ 30 kDa are efficiently eliminated within 1 hr, larger proteins show very long circulation times. To date larger proteins dominate clinical use. For this reason this review shall be focused on larger protein formats above the renal exclusion limit. The importance of proteins is highlighted in the innovation of proteinaceous tumor-targeting systems and personalized medicine. The target specificity achieved with antibodies, in particular antigens whose receptors are overexpressed in tumor cells, allows for accurate drug delivery.

Tumors which develop through large numbers of mutations are often of a heterogeneous phenotype, even among the same cancer types. The advanced development of modern diagnostic and therapeutic techniques has taken advantage of the heterogeneity of tumors to benefit the patient. This is due to the availability of a distinct receptor which can be specifically targeted, through the innovative model of personalized medicine. Human Epidermal Growth Factor Receptor 2 (HER2) is a prime example of this exploitation of heterogeneity. HER2 is overexpressed in 30% of breast cancers [28]. Trastuzumab (Herceptin^®^, Genentech) is a specific HER2 targeting mAb which up-regulates the p27Kip1 protein, resulting in the inhibition of CDK2 and halting cell proliferation [29]. Trastuzumab is the first line treatment for breast cancer, but only in patients with a HER2 overexpression. However, cardiotoxicity associated side effects of trastuzumab occur in approximately a third of patients, therefore screening of patients to determine trastuzumab’s efficacy is of vital importance in risk analysis [30]. This prescreening for the presence of HER2 positive cells allows for a determination of the response of a patient to the drug. Thus, it is possible to distinguish which cancer drugs are most likely to benefit a patient, and then tailor the medication regime to that patient. A current Phase 0 study being run by the NCI, is investigating the use of trastuzumab labeled with 111-indium. By chelating the drug with a linker and the radionuclide, the dose required as a diagnostic agent could be reduced to a clinical dose of 200 μg, compared to the current recommended clinical dose of 2–4 mg/kg, so the safety implications are evident [31,32].

### 5.1. Design of Molecular Imaging Agents Based on Proteins

The specificity and success of radio-labeled proteinaceous drugs depends on both the antibody and the radionuclide chosen. Briefly mentioned before, the half-life of the radionuclide should be approximately equivalent to the drug half-life of the antibody. MAbs have generally long half-lives *in vivo*, on the order of days, due to their large size preventing renal excretion. Furthermore their ability to specifically interact with numerous receptor sites increases their retention time [33]. Thus radionuclides with longer half-lives are preferred with full antibodies. Yet smaller antibodies such as diabodies have a decreased retention time in the body, and can therefore be labeled with shorter lived radionuclides [34]. Any antibody which is selected to be labeled with a radionuclide must be able to withstand radiolysis, as an antibody subjected to a high amount of radioactivity may be altered and lose its immunogenicity [35]. Thus, there is a vital importance in radiolabeled antibodies to ensure that the correct antibody and radiolabel are selected. 

### 5.2. Protein-Based Carrier System

As already described, the high selectivity of antibodies makes them a useful method of targeting in cancer therapy. Targeted antibody therapy was first suggested by Paul Ehrlich when coining the term ‘Magic Bullet’ for a drug which can selectively target its site of action. Creating specific antibodies to markers over-expressed on the surface of solid tumors creates a delivery system capable of mono-targeting tissues possessing this marker. Thus to some extent mAbs are deserving of the title ‘Magic Bullets’. Since their inception mAbs have become increasingly expansive and successful in targeting disease sites [36,37]. Exploiting the specificity of tumor biomarkers allows for the reduction of cytotoxic side-effects associated with radionuclides. The first approved antibody-based drug and diagnostic compounds were murine-based causing the human immune system to recognize them as foreign and eliminate them from the body. Furthermore their low cell penetration and incorrect interaction with the immune system resulted in a poor efficacy. The ability to create recombinant chimeric antibodies was a key advancement in both antibody engineering and developing protein-based carrier systems. By replacing portions of the murine antibody which provoke the immunogenic response, but still preserving the variable binding domains, increased specific binding is attainable. This introduction of human constant domains led to the generation of the first chimeric mAb [33]. There are currently many chimeric antibodies in clinical utility such as rituximab, used in the treatment of CD20 positive B-cell non-Hodgkin’s lymphoma [38]. Additional developments allowed for further decreases in immunogenicity by interchanging the murine hypervariable loops responsible for binding activity with their counter hypervariable loops in the human antibody. This replacement led to the generation of ‘humanized’ antibodies which are even less immunogenic than chimeric mAbs. There are various humanized antibodies approved for medical use, such as bevacizumab, a VEGF antigen used to treat metastatic colorectal and non-small cell lung carcinoma [33]. The engineering of cytotoxic antibodies is simple in theory. By loading the antibody with a radioactive molecule, it is possible to reach relatively high radiation counts at the tumor site. However this high irradiation is possible at a low dose of drug, due to the targeting capabilities of the antibody. The increasing ability to customize antibodies with a wide range of side chains allows for a greater potential for site specific delivery of a radionuclide. This is owing to the functional groups which can be added resulting in an improved targeting to specific receptors [39,40].

mAbs can act in various ways to produce their cytotoxic effects. Many (such as trasutzumab, rituximab [41,42,43]) work together with the parts of the human immune system inducing either antibody-dependent cellular cytotoxity (ADCC) by binding to Fcγ receptor and target cell or complement dependent cytotoxicity [44]. The high sensitivity and specificity attained with mAbs are their greatest features and are therefore a vital requirement for successful therapy. Thus one of the main obstacles of mAb therapy is the heterogeneous distribution of antigen on tumor cells. Additional requirements of mAbs to ensure effective therapy are: fast clearance from blood compartment and sufficient and homogenous tumor penetration [41]. Other than inducing the human immune system, antibodies are also used as carriers of a pharmaceutical to the tumor site. In this use their main function is the transport of radionuclides to the target site. However, the labeled antibody often cannot deliver an appropriate therapeutic dose to the tumor, therefore attachment of more than one radionuclide to the protein has been attempted. However when randomly distributed, a loss of immunoreactivity is caused as seen in the iodine-labeling of IgG antibodies [42]. Antibody-dendrimer conjugates were successfully investigated producing anti-EGFR DOTA derived PAMAM dendrimers [11]. To further increase permeability, attempts in molecular engineering have been made to develop bispecific antibodies with lower molecular weight [45,46].

## 6. Radiolabeling Strategies of Proteins

**Figure 2:**
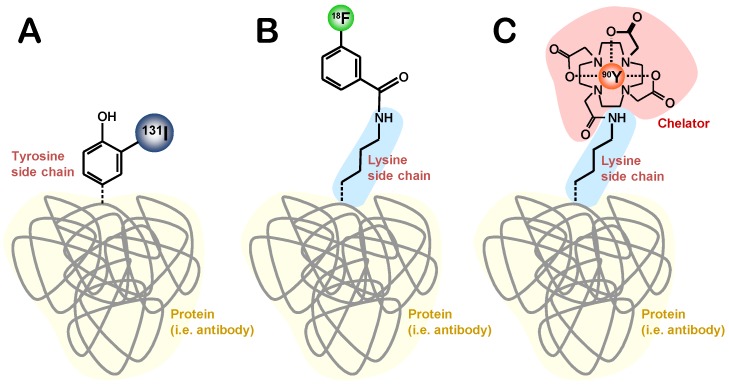
Illustration of the three main strategies for the radiolabeling of proteins. (**A**) direct labeling (**B**) indirect labeling via a prostetic group and (**C**) indirect labeling via complexation. The different methods allow incorporating various types of radiolabels into a protein: (**A**) iodine (**B**) fluorine and (**C**) metallic radionuclides. Maleimides and other functionalities that specifically react with thiols are a common alternative to the use of radiolabeled synthons linked to lysine side chain amines shown in examples (**B**) and (**C**).

There are various radiolabeling strategies available to incorporate a radionuclide into a protein (Figure 2). The choice of technique for a radiochemist depends primarily on the radionuclide used. The radioactive isotopes of iodine possess the ability to be directly integrated into a molecule by electrophilic substitution or indirectly via conjugation. Radioactive metals on the other hand are labeled via complexation with a chelating agent. Many metallic radionuclides possess the ability to form stable complexes with chelating agents, thus allowing for conjugation with a protein. 

### 6.1. Methods of Radiolabelling

#### 6.1.1 Radiohalogenation

##### 6.1.1.1. Radioiodination of Proteins

Radiolabeling molecules with iodine nuclides is of great importance in pharmaceutical radiochemistry. There are over thirty different identified iodine isotopes, but only four are commonly used in radioiodine chemistry: ^123^I, ^124^I, ^125^I and ^131^I. They all possess vital properties, allowing for their application in radiochemistry. 

The differing half-lives of all four allow for a variation in their usages. As seen in Table 2, the most popular use of iodine radionuclides is in molecular imagining providing valuable information on physiological processes. ^123^I is the most suitable radioisotope for molecular imaging and is an ideal choice radionuclide for SPECT imaging. ^131^I can also be used as a γ-emitter for SPECT imaging, however it also is a β-emitter (see Table 1) and is no longer recommended due to its irradiation potential. [47] ^124^I is a positron emitting nuclide, therefore having an application in PET imaging, such as the diagnosis of thyroid cancer [48]. Owing to its long half-life of over 4 days, it could also be utilized in order to monitor physiological processes which occur over long periods. ^124^I has a half-life similar to that of a mAb, therefore making it a key candidate in radioimmunotherapy [49].

**Figure 3:**
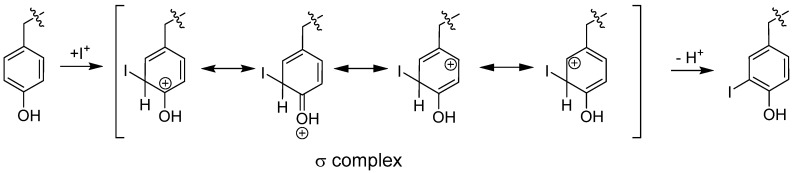
Mechanism of direct electrophilic radioiodination of an iodide electrophile to produce the iodinated product.

The direct radioiodination of a protein is a key method for the synthesis of tumor-targeting radiopharmaceuticals, like ^131^I-labeled rituximab [50] (Table 3). Generally there are two basic approaches of protein radioiodination. The most straightforward approach is direct protein labeling using electrophilic substitution at tyrosine and histidine residues. The radioiodide is oxidized *in situ* creating the electrophile *I^+^. This is done using oxidizing agents like chloramine T, Iodogen^®^ and N-halosuccinimides [51]. The generated electrophile attacks the electron rich of aromatic ring of the amino acid tyrosine, forming a σ-complex [51,52] This substitution is performed at the tyrosine residue due to the electron donating hydroxyl group which stabilizes the σ-complex (see Figure 3). As the labeling of proteins must take place under mild conditions, the attachment of iodine to the tyrosine is highly suitable. 

This straightforward method is performed under mild conditions, which is optimal for the labeling of proteins. Unfortunately, this is only possible when the protein contains accessible tyrosine or histidine residues. A further disadvantage is the accumulation of iodine in the thyroid and stomach when using direct labeling strategies. This is due to the naturally occurring catabolism of proteins by the enzyme tyrosine deiodase, increasing *in vivo* toxicity [53]. Tositumomab (^131^I-B1 mAb) is an anti CD20 antibody conjugated with the mixed β/γ-emitter ^131^I. It received FDA approval for the treatment of Non-Hodgkin’s lymphoma in 2003. This drug is synthesized via direct labeling, using the electrophilic substitution method above with Iodogen^®^. Tositumomab benefits from good response rates, and is still being used in clinical practice [54].

**Figure 4:**
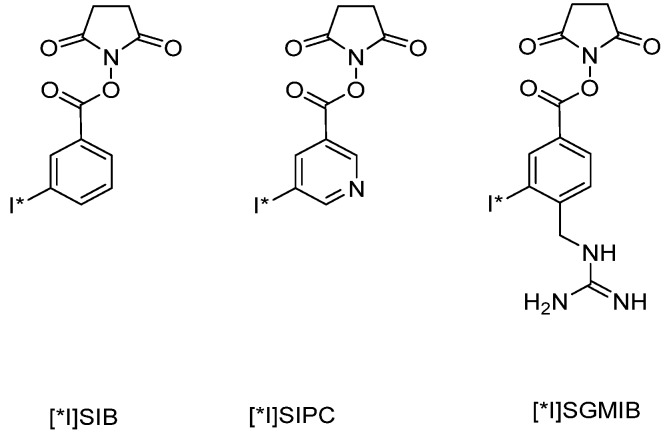
Chemical structures of the chelating agents SIB, SIPC and SGMIB.

However, due to the problems mentioned above, the indirect iodination of proteins via conjugation is a frequently used alternative method. In this approach iodine is incorporated by the application of prosthetic groups containing two functional groups to enable both radioiodination and incorporation to the protein. There are a variety of prosthetic groups used for radioiodination, but the most frequently used are *N*-succinimidyl 5-[*1]iodo-3-pyridinecarboxyl ([^131^I]SIPC) [55] and *N*-succinimidyl-3-[*I]-iodobenzoate ([*I]SIB) [56] (Figure 4).

Both active esters are conjugated to amino groups of the protein and exhibit a high *in vivo* stability. This was successfully shown in the radiolabeling of insulin-attached [^131^I]SIPC, which exhibited a higher *in vivo* stability than directly coupled [^131^I] [53,57]. Another prosthetic group for the acylation of aromatic groups is *N*-succinimidyl-4-guanidinomethyl-3-[I-131]iodobenzbate ([I-131]SGMIB) (Figure 4). It has been successfully applied to internalizing antibodies with a 4-fold increased uptake compared to labeled antibodies with [^131^I]SIPC [58].

##### 6.1.1.2. Protein-Labeling with ^18^F

^18^F, a positron emitter, is often considered the preferred radionuclide for PET imaging due to its many desirable qualities as described above. Similarly to iodination there are two main synthetic strategies to label a protein with ^18^F. The most straightforward method is the direct incorporation of ^18^F into the protein by direct attachment to a tyrosine residue via electrophilic or nucleophilic attacks. ^18^F_2_ is most commonly used in *electrophilic fluorination* due to its high reactivity. Mild conditions are essential for this highly exothermic reaction which must be tightly controlled using low temperatures. The first ^18^F labeled molecules were obtained using this method. However, in modern times this method is less commonly used, due to the lack of regioselectivity leading to the formation of a mixture of products which exhibit low specific activity oft due to an exchange with ^19^F [8,59].

*Nucleophilic fluorination* achieved with fluoride ions is now the most routinely used method. The nucleophile is obtained as an aqueous solution, but due to polar solvation the fluoride is a poor nucleophile. Therefore the fluoride ions are transferred to a non-polar, organic solvent, like DMSO under assistance of phase transfer agents such as [^18^F]KF·K222. Nucleophilic substitution is not possible under mild conditions due to lack of reactivity. Therefore elevated temperatures and basic conditions are used to initiate nucleophilic substitution. However under these harsh conditions most macromolecules, including proteins, are not stable [60,61], so this method is often incompatible with large, temperature sensitive biomolecules such as proteins. 

**Figure 5:**
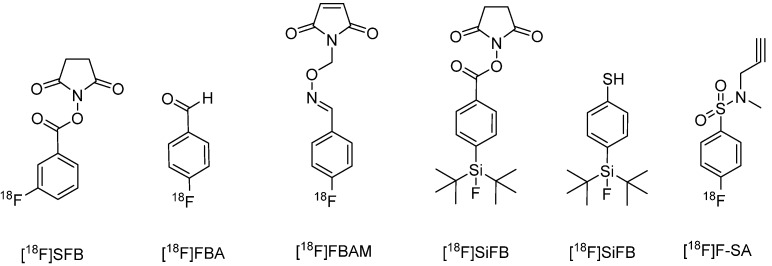
Structures of prosthetic groups used to indirectly incorporate ^18^F proteins.

As an alternative to the direct methods, indirect incorporation of ^18^F can occur via conjugation of ^18^F-labeled reactive precursors (Figure 5). These precursors contain prosthetic groups allowing for the fluoridation and also conjugation with proteins under mild conditions. 

Thus the reaction can take place at room temperature and in aqueous solvents, preferable conditions compared to nucleophilic substitution. The conjugation of the precursors occurs mostly at an amino group on the protein, however can also take place on carboxy or sulfhydryl groups. The attachment of a prosthetic group to an amino group (lysine side chains or the N-terminus of a protein) is carried out with the active ester N-succinimidyl-4-[^18^F]fluorobenzoate ([^18^F]SFB) by alkylation with a good coupling rate of 40%–60% [58]. A disadvantage of using prosthetic groups to incorporate ^18^F is that the formation of prosthetic groups often requires multistep complex methods, likely to result in decreased yields. Some groups have tried to reduce the numerous steps and complexity involved such as the one pot synthesis method suggested by Hou *et al.*, however the yields obtained were poor [62].

In order to decrease the work load, a recent study suggested the use of N-succinimidyl 3-(di-*tert*-butyl[^18^F]fluorosilyl)benzoate ([^18^F]SiFB), a prosthetic group with properties similar to [^18^F]SFB but with a simplified one step radiolabeling procedure [63]. In contrast to direct labeling approaches, indirect labeling with precursors results in the generation of a greater number of radiolabeled molecules containing several modifications and localization and thus varying in structure. The bio-activity of these structures is often difficult to characterize, which has led to the pursuit of a site- specific process producing a homogenous product [64]. The first chemoselective labeling was achieved by Flavell *et al.* in 2008 using [^18^F]fluorobenzaldehyde ([^18^F]FBA). This precursor was used to incorporate an aminooxy functional group into the C terminus of leptin in an aniline-accelerated radiochemical oximation reaction, allowing for unambiguous bioactivity determination [65]. Further site-specific labeling has been obtained by using a silicon-fluoride acceptor (SiFA) reagent such as [^18^F]SiFA-SH. The number of incorporable molecules depends on the number of introduced maleimide groups. In order to preserve the biological activity of the antibody, it is vital that no more than two maleimide groups be introduced [66]. Secondly, the site of modification can affect the biological activity [40]. Further chemoselective labeling can be obtained using an approach based on hydrazone formation. The protein is conjugated with hydrazinonicotinic acid (HYNIC) and successively labeled with [^18^F]fluorobenzaldehyde via hydrazone formation. This approach has been successfully used for the fluorination of human serum albumin [67].

However these chemoselective reactions have drawbacks. The use of appropriate reaction conditions is of utmost importance, with these reactions producing varying yields between 25%–99% [4]. Further difficulties arise when attempting to label proteins containing thiol groups. In order to label proteins containing accessible thiol groups, [^18^F]maleimide reagents, like N-(6-[(4-[^18^F]fluoro-benzylidene)aminooxy]hexyl)maleimide ([^18^F]FBAM) can be used for labeling low-density lipoproteins [64]. The excellent orthogonality of the copper-catalyzed 1,3-dipolar cycloaddition between azides and alkynes yielding triazoles has caused pervasive interest in this reaction to trigger conjugation reactions. This reaction is referred to as click chemistry. In a recent approach 4-[^18^F]fluoro-N-methyl-N-(propyl-2-yn-1-yl)benzenesulfonamide ([^18^F]F-SA) was incorporated into human serum albumin using click chemistry. Ramenda *et al.* successfully demonstrated the promising utility of click chemistry as a reliable method of radiolabelling proteins [61]. The reaction results in the formation of a triazole moiety that has to be considered to influence the properties of conjugates. However, in the case of proteins this relatively small modification is not likely to influence the overall pharmacokinetic properties of large proteins. 

#### 6.1.2. Chelation

The attachment of radionuclides through direct labeling, as explained for iodine and fluorine, is only possible if the nuclides can undergo electrophilic and nucleophilic substitutions. Furthermore these radionuclides must be capable of being attached under physiological conditions due to the heat-labile nature or proteins. 

Direct labeling is thought of as advantageous owing to the simplicity of such a process. However radioactive metal ions are generally more difficult to attach via direct labeling strategies due to their reduced reactivity compared to halogens. Nevertheless there have been direct approaches for labeling proteins with metallic radionuclides. Proteins, such as antibodies, often contain the amino acid cysteine along their amino acid backbone. Cysteine is unique as it is the only natural amino acid containing a thiol group, a key target for complexation with a radionuclide via the formation of a coordinate bond. These thiol groups possess the ability to form disulfide linkages between cysteine groups and are one of the primary mechanisms of polypeptide folding. As such any attempt to label cysteine residues with a radionuclide must first overcome these disulfide cross-linkages. For successful radiolabeling to occur sufficient available thiol groups are necessary to complex with the generated metal radionuclide. Poor site specificity, and lack of stability when coupling with thiol groups are major shortcomings of direct radiolabeling of antibodies with metal nuclides [68]. Approaches to counter this have involved reducing the disulfide linkages to produce thiol groups. However this method if uncontrolled is likely to alter the spatial conformation of the protein [69]. These disadvantages, along with the good chelating properties of metal ions, results in an indirect radiolabeling approach being preferred. These indirect methods link radio-metals to proteins using bifunctional chelators [BFCs]. As their name already indicates, these molecules consists of two functional groups which serve different purposes; one functional group for the binding to the protein and a chelating unit which carries the radionuclide [70,71]. A BFC is essentially made up of three sections, a radionuclide binding unit, a unique ligand framework and a conjugating group for attachment to the antibody [72]. The attachment of metallic radionuclides using chelation is a widely accepted field, with FDA approved radiopharmaceuticals currently in use, like Zevalin^® ^(Spectrum Pharmaceuticals).

Besides the functional requirements, BFCs also have to meet synthetic and chemical requirements. As with prosthetic groups, chelators have to exhibit a high *in vivo* stability in order to ensure patient safety yet also obtain good therapeutic and diagnostic results [73]. To deliver this inertness, BFCs have to be both stable against hydrolysis under physiological conditions and exhibit a strong affinity towards the radionuclide. Furthermore the BFC must be able to withstand radiolysis, as they are used to deliver loads of high radio activity [74]. In addition to those requirements, BFCs are further required to not alter the biological properties and specificity of the protein. Consequently, isomerism is an important characteristic to be considered due to the specific structure of the proteins required for target binding [75].

Owing to the diversity of the metallic radionuclides there is no universal BFC to chelate to all of the radiometals. Therefore many different variations of BFCs are used depending on the choice of radionuclide. The size, charge, and electron configuration of the radiometals will determine the coordination number required of a BFC. The BFC can differ from coordination numbers varying from 2–8 in order to accommodate the radionuclide [76].

##### 6.1.2.1. Acyclic Chelators

The first chelating agents developed for coupling radionuclides to biomolecules were the acyclic chelating agents. These molecules lack a ring system in their coordinating form as compared to macrocyclic chelators which have a preformed ring system. The most common acyclic chelators are derivatives of diethylenetriaminepentaacetic acid (DTPA), which itself was derived from the common metal chelator, ethylenediaminetetraacetic acid (EDTA). EDTA was found initially to lack stability and appropriate coordination values for metallic radionuclides, hence the synthesis of DTPA and its derivatives [77]. Binding of DTPA to proteins, such as an antibody, is possible by the use of the coupling agent isobutyl chloroformate [78,79]. DTPA is capable of binding a wide range of metals including the widely used ^111^In, ^90^Y and ^99m^Tc [60]. Attached to the proteinaceous primary amine, the conjugated acyclic chelating system opens to reveal its octadentate nature. That is, it possesses eight donor sites for interaction with a ligand, therefore DTPA can be considered of hexa- or octa-coordination depending on the radiometal [80]. 

A major advantage of using DTPA analogs as BFCs is the fact that the reactions can occur under mild conditions [81,82]. This is of particular benefit owing to the labile nature of proteins. Reaction conditions and the BFC used must be chosen and scrutinized in order for the antibody to retain its immunogenicity [83]. The DTPA derivative tiuxetan is an example of successful conjugation of a BFC leading to a useful drug molecule. Zevalin^®^ (^90^Y-ibritumomab tiuxetan) therapy uses the mAb ibritumomab, radiolabeled with yttrium and conjugated via the BFC tiuxetan. As stated above, Zevalin^®^ received FDA approval in 2002 for the treatment of Non-Hodgkin’s lymphoma and is currently still being marketed. Compared to the antibody drug rituximab, it shows greater response rates and highlighted the use of anti CD20 radiotherapy [84]. The use of side chains with DTPA derivatives allows for improved binding to proteins, as seen with *p*-isothiocyanatobenzyl diethylenetriaminepentaacetic acid (*p*-SCN-Bz-DTPA). This derivative utilizes an isothiocyanate group, which binds to a free amine on the protein to form a thiourea linkage [85].

**Figure 6:**
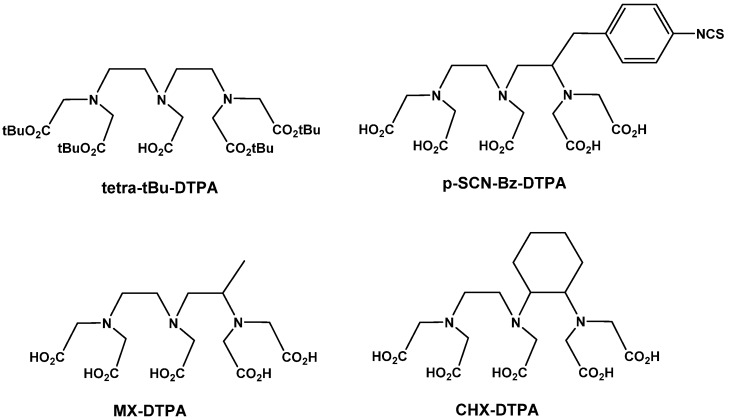
Structures of four DTPA derivatives. Tetra-*t-*Bu-DTPA, containing the protecting groups required to ensure no double substituted products form compared to *p*-SCN-Bz-DTPA which highlights the use of side chains in order to improve binding of the BFC.

Although DTPA has been extensively studied, its low *in vivo* stability diminishes its clinical potential due to dissociation of the radionuclide from the chelating agent. This could cause a particular harm upon radiation sensitive bone marrow [72]. Another negative aspect of conjugation with DTPA is the synthesis of undesired conjugates, such as double substituted DTPA analogues obtained with the DTPA dianhydride. However Hnatowich *et al.* overcame this by using gel chromatography in order to separate the lead product [86]. These negatives have led to the synthesis of DTPA derivatives such as tetra-*t*-Bu-DTPA (see Figure 6) [87] which contains only one free carboxy group, thus eliminating the possibility of forming double substituted DTPA derivatives [88]. An advantage of this BFC is its use in solid phase synthesis owing to its high solubility in a variety of solvents. Another acyclic chelating agent is mercaptoacetyltriglycine (MAG3). This chelating agent is useful at binding ^183^Re and is another alternative to DTPA [89].

**Figure 7:**
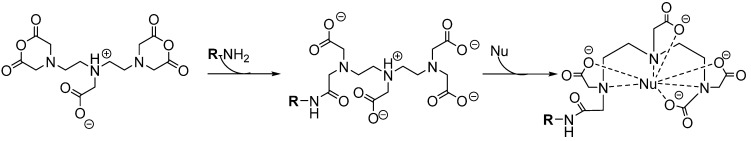
DTPA dianhydride chelating with a metal radionuclide. The mechanism shows the use of a free amine group to open and conjugate to the BFC for attachment of the radionuclide.

However the same radionuclide dissociation issues associated with DTPA are still as problematic in the derivatives of DTPA [60]. The dissociation of the radionuclide from DTPA occurs due to the opening of the chelate ring. Methods and modifications to DTPA have been sought in order to restrict the freedom of motion of this chelate ring and provide an energetic hindrance to the opening of the acyclic backbone [90]. Prevention of this dissociation results in reduced accumulation of radioactive material in the blood stream and increased uptake in the tumor site [91]. This has resulted in the rise of backbone stabilized acyclic chelators which looks to be the way forward for radiolabeling of metals. These acyclic chelators benefit from still having the favorable reaction kinetics associated with DTPA, but at a more appropriate inertness once conjugated. An example of this is cyclohexane-1,2-diamine-*N*,*N*,*N*',*N*'-tetraacetate (CHX-DTPA). CHX-DTPA (Figure 6) contains a *trans*-cyclohexylene bridge which constrains the acyclic chelator by preventing rotation [92]. The radionuclide is stabilized within the chelate ring system resulting in a decreased likelihood of dissociation. Compared to DTPA, CHX-DTPA and the methyl derivative MX-DTPA (Figure 6) benefit from a 2-3 order greater stability when bound to a radioligand [72]. There are two isomeric forms of CHX-DTPA available, CHX-A and CHX-B, with CHX-A possessing greater *in vivo* stability, and is generally preferred in the radiolabeling of mAbs [93,94]. An antibody mimetic aimed at targeting HER2, possessing a single cysteine residue in its amino acid scaffold, has been conjugated to a variant of the maleimido derivative of CHX-A-DTPA. This conjugated ‘affibody’ has been used to provide imaging of high contrast in breast cancer, suggesting that CHX-A-DTPA could have a vital role in molecular imaging and later radiotherapy [95]. Another backbone-stabilized acyclic chelator is mDTPA (Figure 7). This BFC possesses efficient reaction kinetics allowing the reaction to proceed in mild conditions, and deliver DTPA to the antibody in high yield [78].

##### 6.1.2.2. Macrocyclic Chelators

**Figure 8:**
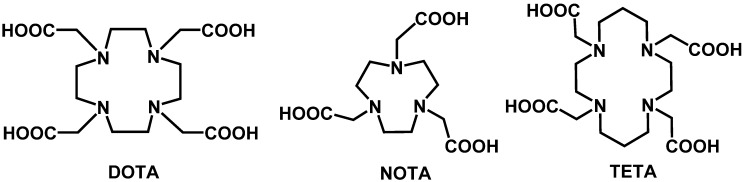
Macrocyclic bifunctional chelating agents: DOTA, NOTA and TETA.

Another strategy to overcome the dissociation problems with bifunctional DTPA derivatives was the search for more stable chelating agents, leading to the use of macrocyclic chelators. Cyclic polyaminoploycarboxylates, also known as macrocyclic chelators, are made up of a tetraza- or triaza- macrocyclic ring. The macrocyclic framework allows for formation of high thermodynamically stable metal complexes with great kinetic inertness. 1,4,7,10-Tetraazacyclododecane-*N,N*'*,N*''*,N*'''-tetraacetic acid (DOTA, Figure 8) is a valuable substitute for DTPA. DOTA and its derivatives can form stable complexes with divalent and trivalent metals and have been used to chelate a wide range of radiometals, including ^111^In, ^86^Y, ^90^Y, ^213^Bi, and ^225^Ac [96]. Radiolabeled DOTA derivatives possess a greater *in vivo* stability than DTPA, owing to their kinetic inertness, a quality which makes them very useful in molecular imaging and radio therapy [97,98]. The ability to secure a radioactive payload is vital for the safety of radiolabeled molecules. However there are also consequences to this beneficial stability. In comparison to DTPA, incorporation of the radiolabel to DOTA occurs slower and with a lower yield. This is thought to be due to poorer formation kinetics, owing to DOTA’s rigid structure, and competition with trace metals [81,98,99]. Labeling of DOTA is a one step process which can be improved by the use of a 4-(2-hydroxyethyl)-1-piperazineethanesulfonic acid (HEPES) or 2-(*N*-morpholino)ethanesulfonic acid (MES) buffer [81,100]. The first approach to conjugate DOTA to a protein was by creating an amide linkage via the activation of one of the carboxy groups in DOTA [90]. However derivatives of DOTA have improved upon this conjugation by using added linker side chains which will bind to the protein. By using X ray crystallography, it is possible to determine which functional groups are not significant in radiolabeled complexes [101]. One such example is the use of an amino acid-substituted BFC, DOTA-Phe-Ala, to bind to an anti EGFR mAb. The amino acid side chain will allow DOTA-Phe-Ala to have a free amine which can be utilized to bind to a wide array of proteins without altering their biological activity [102]. The addition of a side chain also allows for better delivery and targeting properties of the radiolabeled antibody. An example of this are the maleimidocysteineamido-DOTA derivatives which inhibit the release of the BFC-radiometal complex in a pH dependent fashion. This is thought to improve tumor uptake owing to the acidic pH often found in solid tumors, causing an accumulation of radiometal at the tumor site [99].

The ability of DOTA derivatives to complex with ^67^Ga, ^90^Y and ^111^In lends itself for use in radiotherapy. Conjugating a metal radiolabeled DOTA derivative with a protein as a radiotherapeutic is termed antibody guided radiation therapy. Wong *et al.* showed these capabilities by adapting the conjugation of their chimeric anti-CEA antibody to use DOTA instead of the DTPA derivative mentioned above. However the DOTA conjugated antibody had a greater immunogenic host response. This may be due to the chimeric nature of the antibody and not be significantly influenced by the conjugated DOTA [103]. The use of DOTA, however, does not automatically suggest and provide a high level of radionuclide stability in the radiolabel-protein complex. DOTAs ability to complex with ^64^Cu is questionable [104]. As mentioned above, ^64^Cu is a useful radiolabel owing to its appropriate half-life which is similar to that of mAb *in vivo* [105]. However it has a labile nature and when complexed with DOTA it is known to dissociate and accumulate in non-target organs. 1,4,7-Triaza-cyclononane-1,4,7-triacetic acid (NOTA, Figure 8) is a DOTA derivative which has seemingly overcome this problem. It has been widely reported that NOTA has a much better stability with ^64^Cu than DOTA, with less accumulation of the radionuclide appearing in other organs [106,107].

Another common chelating agent is 1,4,8,11-tetraazacyclotetradecane-1,4,8,11-tetraacetic acid (TETA, Figure 8), a macrocyclic BFC primarily used in radiolabeling molecules with ^64^Cu radionuclides. [90,108]. Compared to the acyclic BFCs and DOTA, TETA is a more stable chelating agent when dealing with copper nuclides labeled onto antibodies, in particular the benzyl TETA derivative, as shown by Cole *et al.* [109]. Use of TETA with ^64^Cu in peptides has led to suggestions that TETA could have potential use in the future of molecular imaging of neuroendocrine tumors [110].

## 7. Metallic Nuclide Labeled Pharmaceuticals

### 7.1. ^99m^Tc-Labeled Antibody Radiopharmaceuticals

As stated earlier, ^99m^Tc holds great value in SPECT imaging. There are currently more than 20 discovered isotopes of technetium (Tc), with the most valuable isotope being ^99m^Tc. ^99m^Tc is so useful due to its beneficial nuclear properties, in particular its radioactive half-life of 6.02 h which, together with the excellent purity of the isotope obtained from the ^99^Mo/^99m^Tc generator, allows one to attain the specific radioactivity required for the administration of high radiation counts at a small biological dose. ^99m^Tc is readily available at a low production cost through commercial ^99^Mo/^99m^Tc generators [111]. ^99m^Tc radiolabeled antibodies are becoming of increasing interest due to the targeting capabilities of mAb antibodies. One such example is that of ^99^mTc- labeled mAb girentuximab 250 (mAbG250), an antibody used in the treatment of renal cell carcinoma. By conjugating the antibody with HYNIC and then radiolabeling with ^99m^Tc, a ^99m^Tc-HYNIC-G250 is developed which shows great promise in the radioimmunodetection of renal cell carcinoma [112]. Oosterwijk-Wakka *et al.* furthered this by investigating the potential use of mAbG250 in RIT and also as in combination therapy with tyrosine kinase inhibitors. The potential of this radiolabeled antibody appears to be increasing with positive results in early clinical trials [113]. ^99m^Tc is considered to be a key radiometal for radioimmunoscintigraphy (RIS). ^99m^Tc-MAG3-biotin conjugates have been synthesized and evaluated *in vivo*, to determine their use. Whilst they were unsuitable for RIT due to their excretion via the intestines, the ^99m^Tc-MAG3-biotin conjugates could still find use in RIS [114].

### 7.2. Metallic Radionuclides Other than Tc

Previously we have discussed the different coordination chemistry associated with ^111^In and ^67/68^Ga and the utilization of these radiolabels in molecular imaging when conjugated to peptides [60]. Both of these radionuclides are used in the specific imaging of tumors due to their appropriate range of emissions. As discussed earlier, trastuzumab has successfully been radiolabeled with indium-111. It has been shown that ^111^In-DTPA has been conjugated with trastuzumab in order to determine if it can be used in molecular imaging of HER2 positive breast cancer. Lub-de Hooge *et al.* successfully radiolabeled this antibody which selectively targets the HER2 receptor with high *in vivo* stability [115]. This new radiopharmaceutical is currently in clinical trials. 

^68^Ga is a short lived radionuclide, with a half-life just over an hour (see Table 1). Despite this, its use in immuno-PET imaging has been explored. Diabodies as carriers of radionuclides can be utilized for PET imaging due to their rapid clearance from the blood system even with the short-lived radiometal ^68^Ga [116]. This suggests that radiolabeled diabodies may have prospects in the management of immunotherapy regarding receptor expression of solid tumors [117].

As discussed earlier, ^90^Y is a long range β-emitter and therefore lends itself to use in RIT. Wong *et al.* showed the capabilities of DTPA derivatives as BFCs by use of a chimeric anti-CEA antibody conjugated with ^90^Y isothiocyanatobenzyl DTPA. This yttrium radiolabeled antibody was tested in Phase I clinical trials for the treatment of CEA-producing metastases. This study showed promising results in the large burden tumors but the chimeric nature of the antibody generated some host immunogenic responses [118]. The group then furthered this study by trialing the conjugated antibody in a combination therapy trial with 5-fluorouracil. The results showed that 5-fluorouracil may decrease the immunogenic host response when using this chimeric antibody. This signifies that yttrium radiolabeled proteins may have a future role in combination therapy of tumors [119]. This method was then further enhanced by the use of pretargeting methods as discussed below. Radiolabeling of antibodies with ^90^Y is an avenue well researched as shown by Zevalin^®^, epratuzumab, veltuzumab and clivatuzumab, all featured in the list of antibodies on the market in Table 3. 

Rhenium-188 is another radionuclide used in RIT. Methods to conjugate this metal nuclide includes using the aforementioned chelating agent MAG3. ^188^Re can be produced in a ^188^W/^188^Re generator. It decays via the emission β-particles at a high enough energy, making it appropriate for radiotherapy. After this, ^188^Re then decays further by the emission of gamma photons. This allows for RIT potentially followed by monitoring and analysis of biodistribution [120].

## 8. *In-Vivo* Pretargeting Strategies

A major cause of side-effects of radiolabeled proteins is the accumulation of radioactive materials as a result of the low blood clearance of radiolabeled antibodies. This is especially troublesome in the presence of radiation sensitive bone marrow. To decrease this risk factor, new technologies decouple the targeting of the tumor and the delivery of the radionuclide which improves the blood/tumor ratio, decreasing the irradiation delivered to marrow. Such approaches are known as pretargeting strategies. Despite this being a multi-step process, the advantages prove to be worth the increasing complexity [121,122]. All pretargeting strategies are based on modified bi-specific antibodies which recognize target antigens and can also specifically bind to a second component. Firstly an injection of a large saturating dose of an unlabeled antibody is administered [16]. Then, after sufficient blood clearance of the unlabeled antibody, around two to four days, administration of the radiolabeled effector occurs. This wait is to ensure that adequate unlabeled antibodies have been cleared from the body ensuring that the labeled effector does not bind to circulating antibodies in the blood stream and of sites of low binding affinity, thus reducing unwanted irradiation [121]. Pretargeting is facilitated by the innovative dock and lock technology which allows for the production of many mAB targeting tumor-associated antigens [123]. There are two main approaches using bispecific antibodies: (i) bispecific antihapten antibody-hapten binding [122] and (ii) bispecific antibody conjugated avidin/streptavidin(sAv)-biotin binding. [124]

Improvements have been made to bispecific antibody-hapten binding to enhance the binding affinity. It was discovered that a divalent-hapten molecule would greatly improve tumor uptake. This use of a divalent molecule instead of a monovalent is known as an affinity enhancement system [125,126]. This involves introducing equimolar amounts of Fab’ fragments against the tumor and Fab’ fragments of an anti-DTPA-indium antibody, followed by a bivalent hapten injected once sufficient blood clearance has occurred. This approach is currently used in a preclinical trial of non-Hodgkin’s lymphoma: TF4, an AB fusion protein with two Fabs specific for the CD20 antigen (injected first for tumor targeting). After this, application of ^90^Y-IMP-288 (with 2 histamine-succinyl-glycine residues) improved cure rates compared to therapy of NHL with ^90^Y-veltuzumab [123].

In pretargeting with streptavidin (sAv)-biotin, streptavidin is conjugated to antitumor IgG, followed by an injection of radiolabeled biotin. The biotin molecule is conjugated with a BFC such as DOTA allowing for the introduction of a variety of metallic radiolabels [124,127]. In contrast to bispecific antibody-hapten binding, which utilizes the antibody’s affinity to ensure retention, sAv-biotin benefits from a remarkably high affinity between biotin and sAv. This affinity is due to the four biotin binding arms present on sAv [121]. However as they’re large molecules, IgG and streptavidin reside in the blood stream for a long period of time, and strategies have been implemented to improve the clearance of these molecules. This clearance occurs prior to injection of labeled biotin, to prevent the presence of conjugated sAv and radiolabeled biotin in the circulation. A clearing agent, biotin-galactose-human serum albumin, is injected to remove the non-localized antibody-streptavidin conjugate from blood via the liver [127]. A variation of this coupling can also be implemented by using a multi-step avidin-biotin pretargeting method. As avidin is glycosylated and cleared by the liver, it is possible to decrease the blood circulation concentration of the modified biotin conjugated antibody. Avidin binds to the circulating mAb-biotin complexes and gets cleared by the liver. Then sAv, which is not glycosylated, is administered which upon reaching the tumor site binds to the biotin conjugated IgG selectively. Finally after sufficient clearance of avidin and sAv, radiolabeled biotin is administered which binds to one of the vacant binding arms on the conjugated sAv-biotin-IgG [121]. Phase I – II clinical trials, of post-surgical treatment of stage III-IV glioma, with this avidin-biotin pretargeting approach have produced promising results. Using ^90^Y-biotin after surgery produced a fourfold increased survival rate compared to the control group [128]. The continuation of this study led to suggestions that RIT could be utilized immediately after surgery, and boasted improved survival rates in a cohort of 504 patients [129].

The potential use of pretargeting strategies in tumor therapy has been successfully investigated in preclinical studies, such as the improvement of targeting solid tumors. By targeting the angiogenesis marker, extra domain B fibronectin, with an antibody hapten system labeled with ^111^In gave a three-fold improvement compared to non-targeted endoradiotherapy [130]. Pre-targeting can also improve molecular imaging [122]. Another benefit with this increased selectivity and tumor retention is the possibility to administer larger radioactive quantities to the tumor. Up to five times greater activity is obtainable while also maintaining a low radiation concentration in the bloodstream [16].

## 9. Conclusions

Vaidyanathan *et al.* are currently investigating the use of trifunctional chelating agents. [130] That is, a chelating agent which binds to the antibody as normal but has two groups to bind to radiolabels. The benefit of this is that it would allow the delivery of two different halogens, radiometals or a combination of the two. This could prove vitally important as it would allow for an α- and a β-emitter to be delivered to the same tumor. Thus the long range capabilities of the β-emitter would combine with the devastating effects of α-particulates. Currently a SIB-DOTA prosthetic group is under investigation. This chelating agent allows for the radiolabeling of a halogen and a metal within the same molecule. [131] The potential of delivering a mixture of radiolabels, each with their own unique properties, may be of great benefit in the future of RIT.

As discussed in this paper, antibodies are large macromolecules which possess all the features you would expect. The innovation of nanobodies, could overcome the long half-lives and poor uptake associated with these proteinaceous drugs. Above we have discussed diabodies, however nanobodies is a step further with the antibody fragment containing only the variable region, responsible for the binding ability of mAbs. Use of nanobodies would provide rapid uptake into tumors and short clearance times, improving patient safety. [132,133] Affibodies, chemically accessible peptides with antibody-like properties, consist of a 58 amino acid scaffold derived from the Z domain of the staphylococcal protein A. [134] Due to their favorable *in vivo* properties this class of molecules allows to develop small molecules with excellent targeting properties. [135] The use of α-emitters in α-therapy poses a potential outlet for α-therapy. Currently the radionuclides bismuth-213 and actinium-225 are being investigated for therapeutic application. Combining this with an antibody, would allow for specific targeting, delivering the alpha emitter selectively to the tumor, improving safety. However, the half-life of ^213^Bi is 45 min, not ideal for antibody conjugation. ^225^Ac on the other hand has a half-life of 1 week, much more suitable for antibody therapy. Thus combining α-therapy with proteinaceous targeting systems may provide future benefit in the treatment of tumors [136]. 
